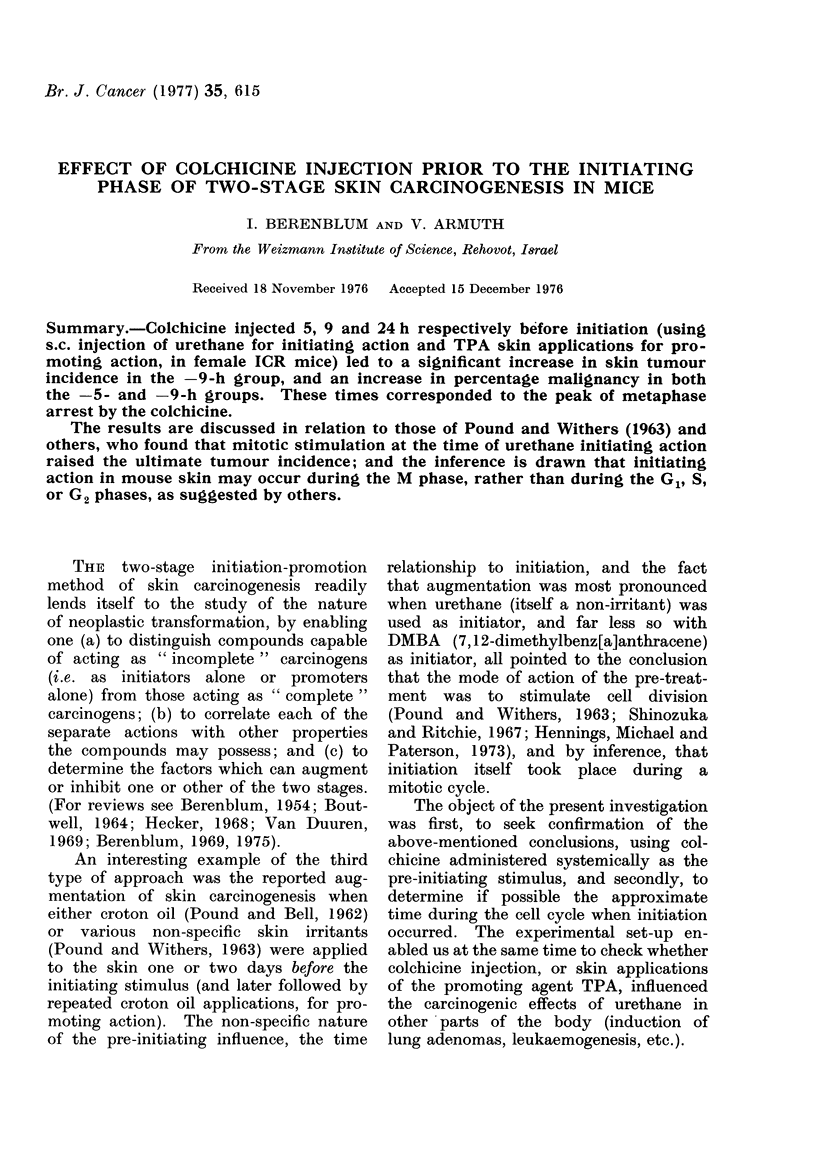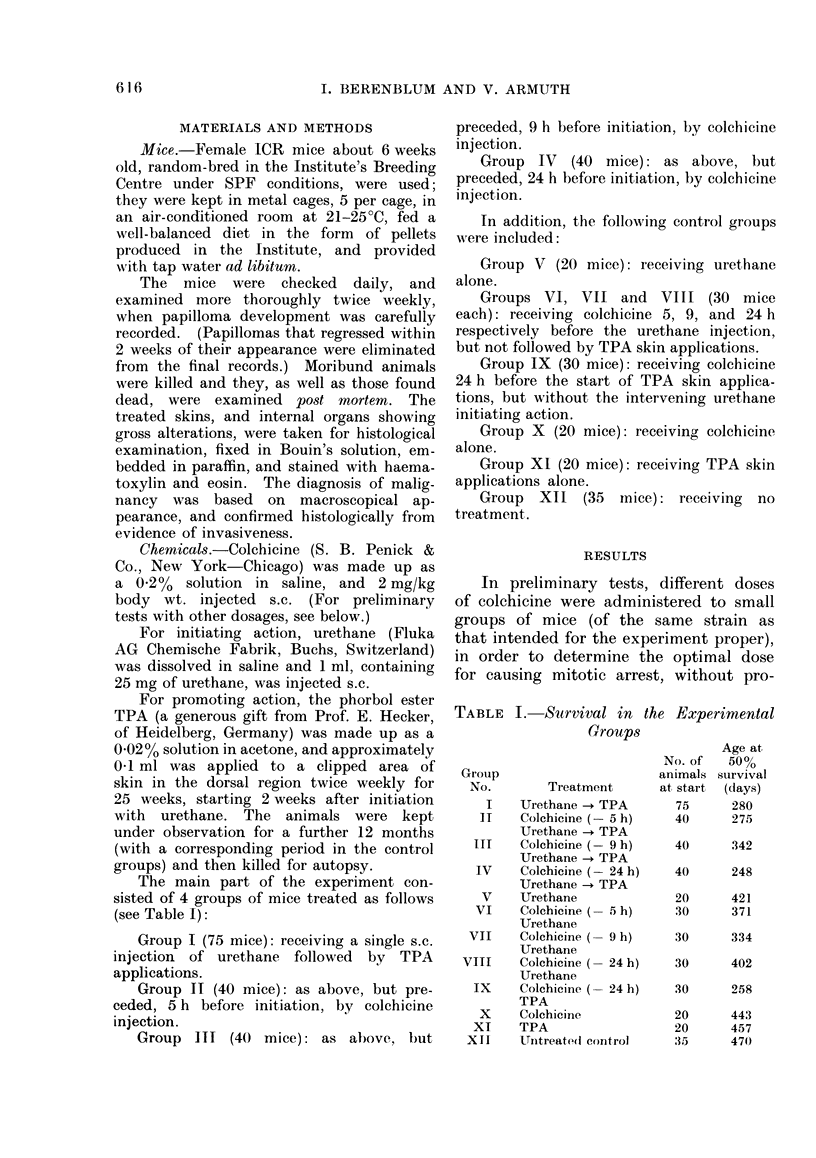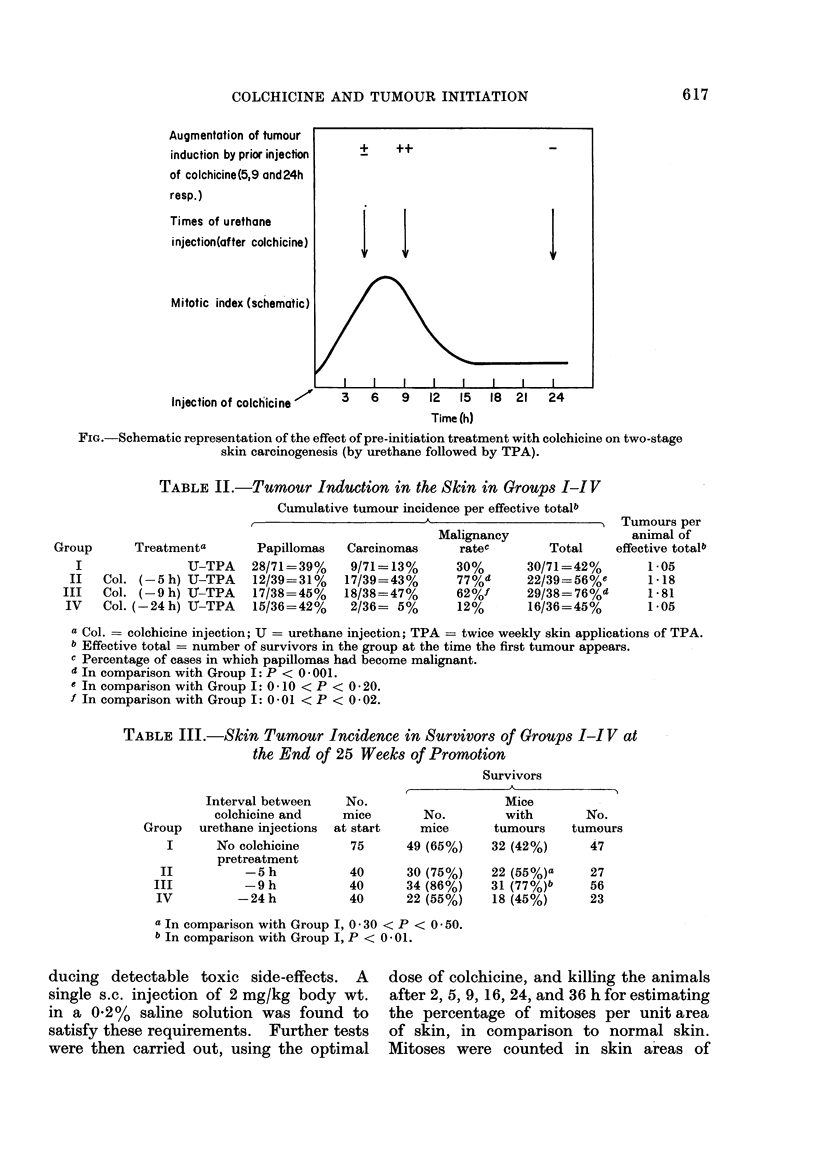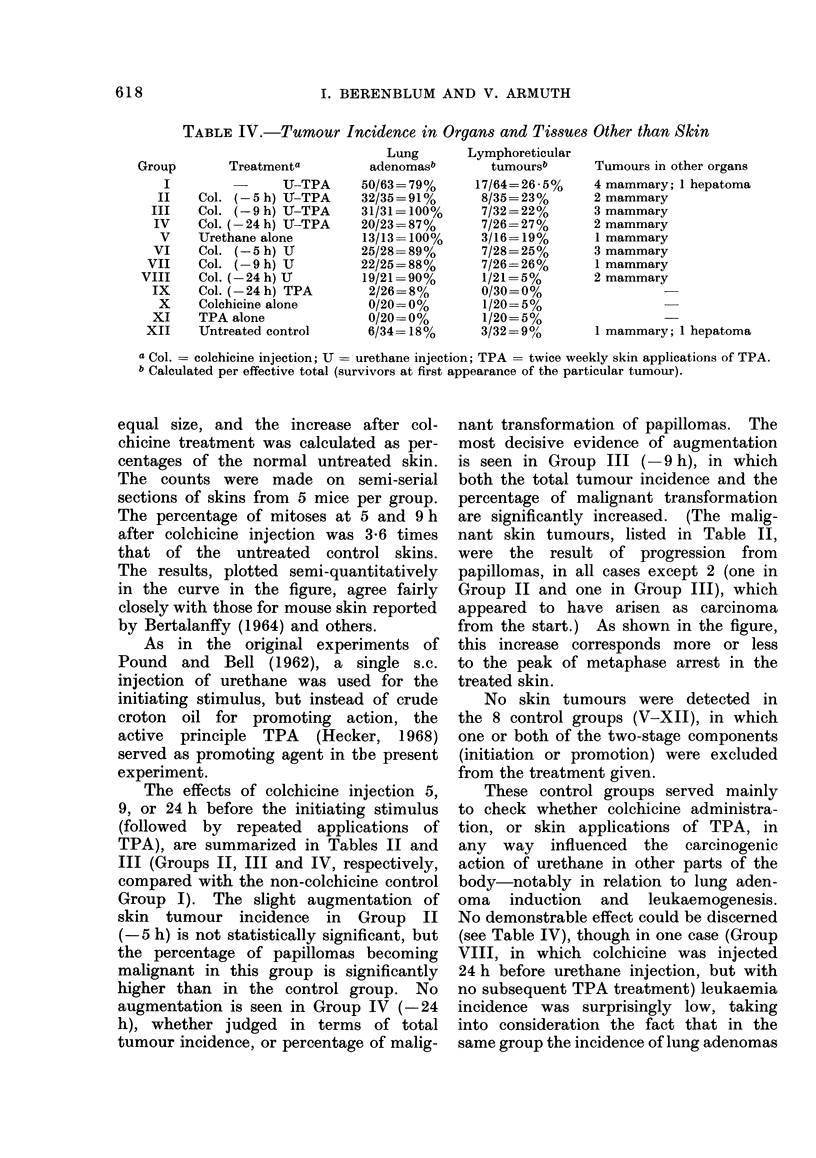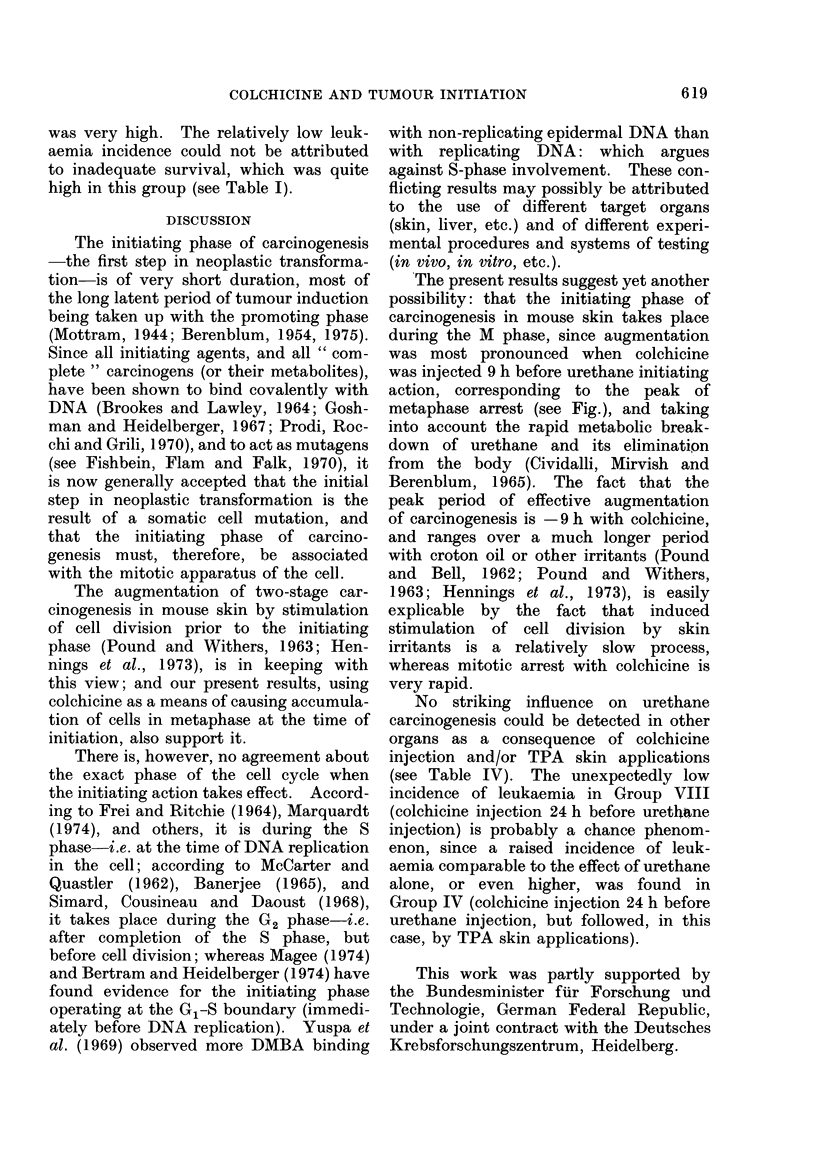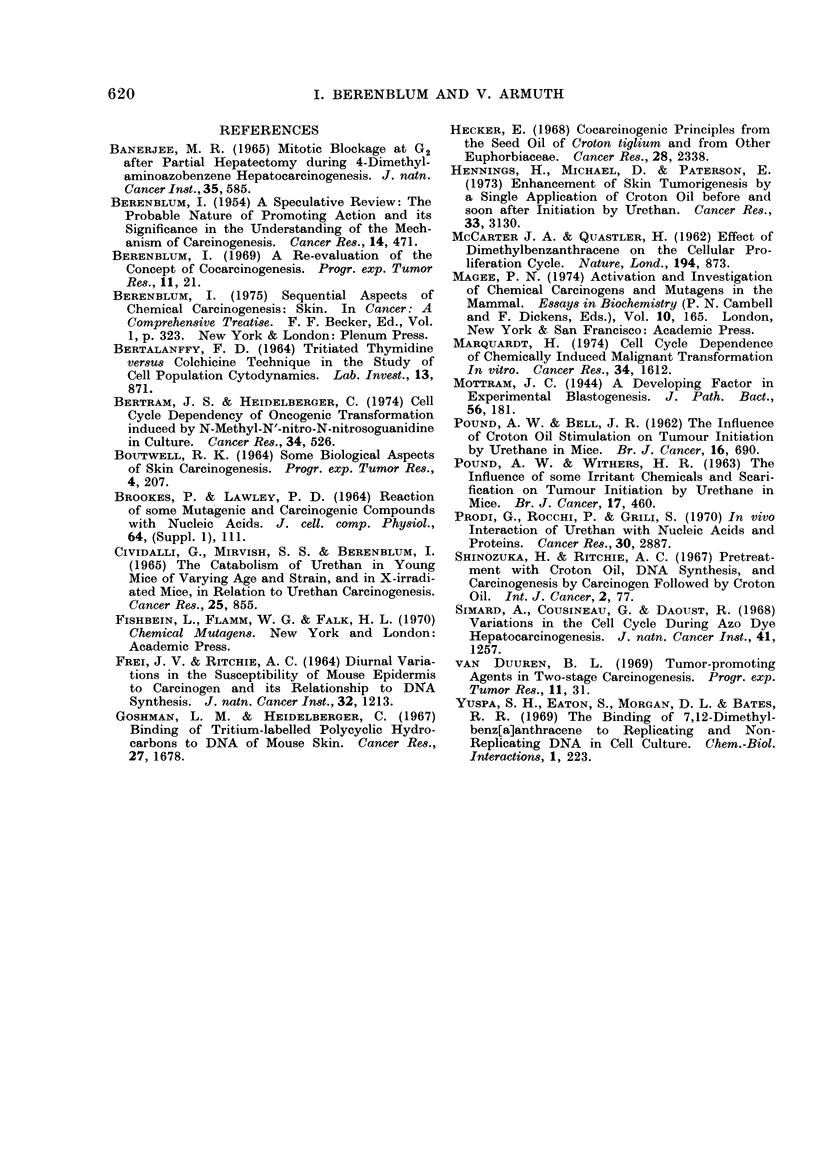# Effect of colchicine injection prior to the initiating phase of two-stage skin carcinogenesis in mice.

**DOI:** 10.1038/bjc.1977.96

**Published:** 1977-05

**Authors:** I. Berenblum, V. Armuth

## Abstract

Colchicine injected 5, 9 and 24 h respectively before initiation (using s.c. injection of urethane for initiating action and TPA skin applications for promoting action, in female ICR mice) led to a significant increase in skin tumour incidence in the --9-h group, and an increase in percentage malignancy in both the --5- and --9-h groups. These times corresponded to the peak of metaphase arrest by the colchicine. The results are discussed in relation in those of Pound and Withers (1963) and others, who found that mitotic stimulation at the time of urethane initiating action raised the ultimate tumour incidence; and the inference is drawn that initiating action in mouse skin may occur during the M phase, rather than during the G1, S, or G2 phases, as suggested by others.


*Br. J. Cancer* (1977) **35**, 615

### EFFECT OF COLCHICINE INJECTION PRIOR TO THE INITIATING PHASE OF TWO-STAGE SKIN CARCINOGENESIS IN MICE

I. BERENBLUM AND V. ARMUTH

*From the Weizmann Institute of Science, Rehovot, Israel*

Received 18 November 1976 Accepted 15 December 1976

**Summary.—Colchicine injected 5, 9 and 24 h respectively before initiation (using s.c. injection of urethane for initiating action and TPA skin applications for promoting action, in female ICR mice) led to a significant increase in skin tumour incidence in the −9-h group, and an increase in percentage malignancy in both the −5- and −9-h groups. These times corresponded to the peak of metaphase arrest by the colchicine.**

**The results are discussed in relation to those of Pound and Withers (1963) and others, who found that mitotic stimulation at the time of urethane initiating action raised the ultimate tumour incidence; and the inference is drawn that initiating action in mouse skin may occur during the M phase, rather than during the $G_1$, S, or $G_2$ phases, as suggested by others.**

THE two-stage initiation-promotion method of skin carcinogenesis readily lends itself to the study of the nature of neoplastic transformation, by enabling one (a) to distinguish compounds capable of acting as " incomplete " carcinogens (*i.e.* as initiators alone or promoters alone) from those acting as " complete " carcinogens; (b) to correlate each of the separate actions with other properties the compounds may possess; and (c) to determine the factors which can augment or inhibit one or other of the two stages. (For reviews see Berenblum, 1954; Boutwell, 1964; Hecker, 1968; Van Duuren, 1969; Berenblum, 1969, 1975).

An interesting example of the third type of approach was the reported augmentation of skin carcinogenesis when either croton oil (Pound and Bell, 1962) or various non-specific skin irritants (Pound and Withers, 1963) were applied to the skin one or two days *before* the initiating stimulus (and later followed by repeated croton oil applications, for promoting action). The non-specific nature of the pre-initiating influence, the time relationship to initiation, and the fact that augmentation was most pronounced when urethane (itself a non-irritant) was used as initiator, and far less so with DMBA (7,12-dimethylbenz[a]anthracene) as initiator, all pointed to the conclusion that the mode of action of the pre-treatment was to stimulate cell division (Pound and Withers, 1963; Shinozuka and Ritchie, 1967; Hennings, Michael and Paterson, 1973), and by inference, that initiation itself took place during a mitotic cycle.

The object of the present investigation was first, to seek confirmation of the above-mentioned conclusions, using colchicine administered systemically as the pre-initiating stimulus, and secondly, to determine if possible the approximate time during the cell cycle when initiation occurred. The experimental set-up enabled us at the same time to check whether colchicine injection, or skin applications of the promoting agent TPA, influenced the carcinogenic effects of urethane in other parts of the body (induction of lung adenomas, leukaemogenesis, etc.).

### MATERIALS AND METHODS

*Mice.*—Female ICR mice about 6 weeks old, random-bred in the Institute's Breeding Centre under SPF conditions, were used; they were kept in metal cages, 5 per cage, in an air-conditioned room at 21–25°C, fed a well-balanced diet in the form of pellets produced in the Institute, and provided with tap water *ad libitum.*

The mice were checked daily, and examined more thoroughly twice weekly, when papilloma development was carefully recorded. (Papillomas that regressed within 2 weeks of their appearance were eliminated from the final records.) Moribund animals were killed and they, as well as those found dead, were examined *post mortem.* The treated skins, and internal organs showing gross alterations, were taken for histological examination, fixed in Bouin's solution, embedded in paraffin, and stained with haematoxylin and eosin. The diagnosis of malignancy was based on macroscopical appearance, and confirmed histologically from evidence of invasiveness.

*Chemicals.*—Colchicine (S. B. Penick & Co., New York—Chicago) was made up as a 0·2% solution in saline, and 2 mg/kg body wt. injected s.c. (For preliminary tests with other dosages, see below.)

For initiating action, urethane (Fluka AG Chemische Fabrik, Buchs, Switzerland) was dissolved in saline and 1 ml, containing 25 mg of urethane, was injected s.c.

For promoting action, the phorbol ester TPA (a generous gift from Prof. E. Hecker, of Heidelberg, Germany) was made up as a 0·02% solution in acetone, and approximately 0·1 ml was applied to a clipped area of skin in the dorsal region twice weekly for 25 weeks, starting 2 weeks after initiation with urethane. The animals were kept under observation for a further 12 months (with a corresponding period in the control groups) and then killed for autopsy.

The main part of the experiment consisted of 4 groups of mice treated as follows (see Table I):

Group I (75 mice): receiving a single s.c. injection of urethane followed by TPA applications.

Group II (40 mice): as above, but preceded, 5 h before initiation, by colchicine injection.

Group III (40 mice): as above, but preceded, 9 h before initiation, by colchicine injection.

Group IV (40 mice): as above, but preceded, 24 h before initiation, by colchicine injection.

In addition, the following control groups were included:

Group V (20 mice): receiving urethane alone.

Groups VI, VII and VIII (30 mice each): receiving colchicine 5, 9, and 24 h respectively before the urethane injection, but not followed by TPA skin applications.

Group IX (30 mice): receiving colchicine 24 h before the start of TPA skin applications, but without the intervening urethane initiating action.

Group X (20 mice): receiving colchicine alone.

Group XI (20 mice): receiving TPA skin applications alone.

Group XII (35 mice): receiving no treatment.

### RESULTS

In preliminary tests, different doses of colchicine were administered to small groups of mice (of the same strain as that intended for the experiment proper), in order to determine the optimal dose for causing mitotic arrest, without pro-

TABLE I.—*Survival in the Experimental Groups*

| Group No. | Treatment | No. of animals at start | Age at 50% survival (days) |
|---|---|---|---|
| I | Urethane → TPA | 75 | 280 |
| II | Colchicine (− 5 h) Urethane → TPA | 40 | 275 |
| III | Colchicine (− 9 h) Urethane → TPA | 40 | 342 |
| IV | Colchicine (− 24 h) Urethane → TPA | 40 | 248 |
| V | Urethane | 20 | 421 |
| VI | Colchicine (− 5 h) Urethane | 30 | 371 |
| VII | Colchicine (− 9 h) Urethane | 30 | 334 |
| VIII | Colchicine (− 24 h) Urethane | 30 | 402 |
| IX | Colchicine (− 24 h) TPA | 30 | 258 |
| X | Colchicine | 20 | 443 |
| XI | TPA | 20 | 457 |
| XII | Untreated control | 35 | 470 |

FIG.—Schematic representation of the effect of pre-initiation treatment with colchicine on two-stage skin carcinogenesis (by urethane followed by TPA).

TABLE II.—*Tumour Induction in the Skin in Groups I–IV*

| Group | Treatment[a] | Cumulative tumour incidence per effective total[b]: Papillomas | Carcinomas | Malignancy rate[c] | Total | Tumours per animal of effective total[b] |
|---|---|---|---|---|---|---|
| I | U–TPA | 28/71=39% | 9/71=13% | 30% | 30/71=42% | 1·05 |
| II | Col. (−5 h) U–TPA | 12/39=31% | 17/39=43% | 77%[d] | 22/39=56%[e] | 1·18 |
| III | Col. (−9 h) U–TPA | 17/38=45% | 18/38=47% | 62%[f] | 29/38=76%[d] | 1·81 |
| IV | Col. (−24 h) U–TPA | 15/36=42% | 2/36= 5% | 12% | 16/36=45% | 1·05 |

[a] Col. = colchicine injection; U = urethane injection; TPA = twice weekly skin applications of TPA.
[b] Effective total = number of survivors in the group at the time the first tumour appears.
[c] Percentage of cases in which papillomas had become malignant.
[d] In comparison with Group I: $P < 0{\cdot}001$.
[e] In comparison with Group I: $0{\cdot}10 < P < 0{\cdot}20$.
[f] In comparison with Group I: $0{\cdot}01 < P < 0{\cdot}02$.

TABLE III.—*Skin Tumour Incidence in Survivors of Groups I–IV at the End of 25 Weeks of Promotion*

| Group | Interval between colchicine and urethane injections | No. mice at start | Survivors: No. mice | Mice with tumours | No. tumours |
|---|---|---|---|---|---|
| I | No colchicine pretreatment | 75 | 49 (65%) | 32 (42%) | 47 |
| II | −5 h | 40 | 30 (75%) | 22 (55%)[a] | 27 |
| III | −9 h | 40 | 34 (86%) | 31 (77%)[b] | 56 |
| IV | −24 h | 40 | 22 (55%) | 18 (45%) | 23 |

[a] In comparison with Group I, $0{\cdot}30 < P < 0{\cdot}50$.
[b] In comparison with Group I, $P < 0{\cdot}01$.

ducing detectable toxic side-effects. A single s.c. injection of 2 mg/kg body wt. in a 0·2% saline solution was found to satisfy these requirements. Further tests were then carried out, using the optimal dose of colchicine, and killing the animals after 2, 5, 9, 16, 24, and 36 h for estimating the percentage of mitoses per unit area of skin, in comparison to normal skin. Mitoses were counted in skin areas of

TABLE IV.—*Tumour Incidence in Organs and Tissues Other than Skin*

| Group | Treatment[a] | Lung adenomas[b] | Lymphoreticular tumours[b] | Tumours in other organs |
|---|---|---|---|---|
| I | — U–TPA | 50/63 = 79% | 17/64 = 26·5% | 4 mammary; 1 hepatoma |
| II | Col. (−5 h) U–TPA | 32/35 = 91% | 8/35 = 23% | 2 mammary |
| III | Col. (−9 h) U–TPA | 31/31 = 100% | 7/32 = 22% | 3 mammary |
| IV | Col. (−24 h) U–TPA | 20/23 = 87% | 7/26 = 27% | 2 mammary |
| V | Urethane alone | 13/13 = 100% | 3/16 = 19% | 1 mammary |
| VI | Col. (−5 h) U | 25/28 = 89% | 7/28 = 25% | 3 mammary |
| VII | Col. (−9 h) U | 22/25 = 88% | 7/26 = 26% | 1 mammary |
| VIII | Col. (−24 h) U | 19/21 = 90% | 1/21 = 5% | 2 mammary |
| IX | Col. (−24 h) TPA | 2/26 = 8% | 0/30 = 0% | — |
| X | Colchicine alone | 0/20 = 0% | 1/20 = 5% | — |
| XI | TPA alone | 0/20 = 0% | 1/20 = 5% | — |
| XII | Untreated control | 6/34 = 18% | 3/32 = 9% | 1 mammary; 1 hepatoma |

[a] Col. = colchicine injection; U = urethane injection; TPA = twice weekly skin applications of TPA.
[b] Calculated per effective total (survivors at first appearance of the particular tumour).

equal size, and the increase after colchicine treatment was calculated as percentages of the normal untreated skin. The counts were made on semi-serial sections of skins from 5 mice per group. The percentage of mitoses at 5 and 9 h after colchicine injection was 3·6 times that of the untreated control skins. The results, plotted semi-quantitatively in the curve in the figure, agree fairly closely with those for mouse skin reported by Bertalanffy (1964) and others.

As in the original experiments of Pound and Bell (1962), a single s.c. injection of urethane was used for the initiating stimulus, but instead of crude croton oil for promoting action, the active principle TPA (Hecker, 1968) served as promoting agent in tbe present experiment.

The effects of colchicine injection 5, 9, or 24 h before the initiating stimulus (followed by repeated applications of TPA), are summarized in Tables II and III (Groups II, III and IV, respectively, compared with the non-colchicine control Group I). The slight augmentation of skin tumour incidence in Group II (—5 h) is not statistically significant, but the percentage of papillomas becoming malignant in this group is significantly higher than in the control group. No augmentation is seen in Group IV (—24 h), whether judged in terms of total tumour incidence, or percentage of malignant transformation of papillomas. The most decisive evidence of augmentation is seen in Group III (—9 h), in which both the total tumour incidence and the percentage of malignant transformation are significantly increased. (The malignant skin tumours, listed in Table II, were the result of progression from papillomas, in all cases except 2 (one in Group II and one in Group III), which appeared to have arisen as carcinoma from the start.) As shown in the figure, this increase corresponds more or less to the peak of metaphase arrest in the treated skin.

No skin tumours were detected in the 8 control groups (V–XII), in which one or both of the two-stage components (initiation or promotion) were excluded from the treatment given.

These control groups served mainly to check whether colchicine administration, or skin applications of TPA, in any way influenced the carcinogenic action of urethane in other parts of the body—notably in relation to lung adenoma induction and leukaemogenesis. No demonstrable effect could be discerned (see Table IV), though in one case (Group VIII, in which colchicine was injected 24 h before urethane injection, but with no subsequent TPA treatment) leukaemia incidence was surprisingly low, taking into consideration the fact that in the same group the incidence of lung adenomas was very high. The relatively low leukaemia incidence could not be attributed to inadequate survival, which was quite high in this group (see Table I).

### DISCUSSION

The initiating phase of carcinogenesis—the first step in neoplastic transformation—is of very short duration, most of the long latent period of tumour induction being taken up with the promoting phase (Mottram, 1944; Berenblum, 1954, 1975). Since all initiating agents, and all " complete " carcinogens (or their metabolites), have been shown to bind covalently with DNA (Brookes and Lawley, 1964; Goshman and Heidelberger, 1967; Prodi, Rocchi and Grili, 1970), and to act as mutagens (see Fishbein, Flam and Falk, 1970), it is now generally accepted that the initial step in neoplastic transformation is the result of a somatic cell mutation, and that the initiating phase of carcinogenesis must, therefore, be associated with the mitotic apparatus of the cell.

The augmentation of two-stage carcinogenesis in mouse skin by stimulation of cell division prior to the initiating phase (Pound and Withers, 1963; Hennings *et al.*, 1973), is in keeping with this view; and our present results, using colchicine as a means of causing accumulation of cells in metaphase at the time of initiation, also support it.

There is, however, no agreement about the exact phase of the cell cycle when the initiating action takes effect. According to Frei and Ritchie (1964), Marquardt (1974), and others, it is during the S phase—*i.e.* at the time of DNA replication in the cell; according to McCarter and Quastler (1962), Banerjee (1965), and Simard, Cousineau and Daoust (1968), it takes place during the $G_2$ phase—*i.e.* after completion of the S phase, but before cell division; whereas Magee (1974) and Bertram and Heidelberger (1974) have found evidence for the initiating phase operating at the $G_1$-S boundary (immediately before DNA replication). Yuspa *et al.* (1969) observed more DMBA binding with non-replicating epidermal DNA than with replicating DNA: which argues against S-phase involvement. These conflicting results may possibly be attributed to the use of different target organs (skin, liver, etc.) and of different experimental procedures and systems of testing (*in vivo*, *in vitro*, etc.).

The present results suggest yet another possibility: that the initiating phase of carcinogenesis in mouse skin takes place during the M phase, since augmentation was most pronounced when colchicine was injected 9 h before urethane initiating action, corresponding to the peak of metaphase arrest (see Fig.), and taking into account the rapid metabolic breakdown of urethane and its elimination from the body (Cividalli, Mirvish and Berenblum, 1965). The fact that the peak period of effective augmentation of carcinogenesis is $-9$ h with colchicine, and ranges over a much longer period with croton oil or other irritants (Pound and Bell, 1962; Pound and Withers, 1963; Hennings *et al.*, 1973), is easily explicable by the fact that induced stimulation of cell division by skin irritants is a relatively slow process, whereas mitotic arrest with colchicine is very rapid.

No striking influence on urethane carcinogenesis could be detected in other organs as a consequence of colchicine injection and/or TPA skin applications (see Table IV). The unexpectedly low incidence of leukaemia in Group VIII (colchicine injection 24 h before urethane injection) is probably a chance phenomenon, since a raised incidence of leukaemia comparable to the effect of urethane alone, or even higher, was found in Group IV (colchicine injection 24 h before urethane injection, but followed, in this case, by TPA skin applications).

This work was partly supported by the Bundesminister für Forschung und Technologie, German Federal Republic, under a joint contract with the Deutsches Krebsforschungszentrum, Heidelberg.

### REFERENCES

BANERJEE, M. R. (1965) Mitotic Blockage at $G_2$ after Partial Hepatectomy during 4-Dimethylaminoazobenzene Hepatocarcinogenesis. *J. natn. Cancer Inst.*, **35**, 585.

BERENBLUM, I. (1954) A Speculative Review: The Probable Nature of Promoting Action and its Significance in the Understanding of the Mechanism of Carcinogenesis. *Cancer Res.*, **14**, 471.

BERENBLUM, I. (1969) A Re-evaluation of the Concept of Cocarcinogenesis. *Progr. exp. Tumor Res.*, **11**, 21.

BERENBLUM, I. (1975) Sequential Aspects of Chemical Carcinogenesis: Skin. In *Cancer: A Comprehensive Treatise*. F. F. Becker, Ed., Vol. 1, p. 323. New York & London: Plenum Press.

BERTALANFFY, F. D. (1964) Tritiated Thymidine *versus* Colchicine Technique in the Study of Cell Population Cytodynamics. *Lab. Invest.*, **13**, 871.

BERTRAM, J. S. & HEIDELBERGER, C. (1974) Cell Cycle Dependency of Oncogenic Transformation induced by N-Methyl-N′-nitro-N-nitrosoguanidine in Culture. *Cancer Res.*, **34**, 526.

BOUTWELL, R. K. (1964) Some Biological Aspects of Skin Carcinogenesis. *Progr. exp. Tumor Res.*, **4**, 207.

BROOKES, P. & LAWLEY, P. D. (1964) Reaction of some Mutagenic and Carcinogenic Compounds with Nucleic Acids. *J. cell. comp. Physiol.*, **64**, (Suppl. 1), 111.

CIVIDALLI, G., MIRVISH, S. S. & BERENBLUM, I. (1965) The Catabolism of Urethan in Young Mice of Varying Age and Strain, and in X-irradiated Mice, in Relation to Urethan Carcinogenesis. *Cancer Res.*, **25**, 855.

FISHBEIN, L., FLAMM, W. G. & FALK, H. L. (1970) *Chemical Mutagens*. New York and London: Academic Press.

FREI, J. V. & RITCHIE, A. C. (1964) Diurnal Variations in the Susceptibility of Mouse Epidermis to Carcinogen and its Relationship to DNA Synthesis. *J. natn. Cancer Inst.*, **32**, 1213.

GOSHMAN, L. M. & HEIDELBERGER, C. (1967) Binding of Tritium-labelled Polycyclic Hydrocarbons to DNA of Mouse Skin. *Cancer Res.*, **27**, 1678.

HECKER, E. (1968) Cocarcinogenic Principles from the Seed Oil of *Croton tiglium* and from Other Euphorbiaceae. *Cancer Res.*, **28**, 2338.

HENNINGS, H., MICHAEL, D. & PATERSON, E. (1973) Enhancement of Skin Tumorigenesis by a Single Application of Croton Oil before and soon after Initiation by Urethan. *Cancer Res.*, **33**, 3130.

McCARTER J. A. & QUASTLER, H. (1962) Effect of Dimethylbenzanthracene on the Cellular Proliferation Cycle. *Nature, Lond.*, **194**, 873.

MAGEE, P. N. (1974) Activation and Investigation of Chemical Carcinogens and Mutagens in the Mammal. *Essays in Biochemistry* (P. N. Cambell and F. Dickens, Eds.), Vol. **10**, 165. London, New York & San Francisco: Academic Press.

MARQUARDT, H. (1974) Cell Cycle Dependence of Chemically Induced Malignant Transformation *In vitro*. *Cancer Res.*, **34**, 1612.

MOTTRAM, J. C. (1944) A Developing Factor in Experimental Blastogenesis. *J. Path. Bact.*, **56**, 181.

POUND, A. W. & BELL, J. R. (1962) The Influence of Croton Oil Stimulation on Tumour Initiation by Urethane in Mice. *Br. J. Cancer*, **16**, 690.

POUND, A. W. & WITHERS, H. R. (1963) The Influence of some Irritant Chemicals and Scarification on Tumour Initiation by Urethane in Mice. *Br. J. Cancer*, **17**, 460.

PRODI, G., ROCCHI, P. & GRILI, S. (1970) *In vivo* Interaction of Urethan with Nucleic Acids and Proteins. *Cancer Res.*, **30**, 2887.

SHINOZUKA, H. & RITCHIE, A. C. (1967) Pretreatment with Croton Oil, DNA Synthesis, and Carcinogenesis by Carcinogen Followed by Croton Oil. *Int. J. Cancer*, **2**, 77.

SIMARD, A., COUSINEAU, G. & DAOUST, R. (1968) Variations in the Cell Cycle During Azo Dye Hepatocarcinogenesis. *J. natn. Cancer Inst.*, **41**, 1257.

VAN DUUREN, B. L. (1969) Tumor-promoting Agents in Two-stage Carcinogenesis. *Progr. exp. Tumor Res.*, **11**, 31.

YUSPA, S. H., EATON, S., MORGAN, D. L. & BATES, R. R. (1969) The Binding of 7,12-Dimethylbenz[a]anthracene to Replicating and Non-Replicating DNA in Cell Culture. *Chem.-Biol. Interactions*, **1**, 223.